# Clinical validity of increased cortical binding of tau ligands of the THK family and PBB3 on PET as biomarkers for Alzheimer’s disease in the context of a structured 5-phase development framework

**DOI:** 10.1007/s00259-021-05277-4

**Published:** 2021-03-15

**Authors:** Konstantinos Chiotis, Alessandra Dodich, Marina Boccardi, Cristina Festari, Alexander Drzezga, Oskar Hansson, Rik Ossenkoppele, Giovanni Frisoni, Valentina Garibotto, Agneta Nordberg

**Affiliations:** 1 Nordberg Translational Molecular Imaging Lab, Division of Clinical Geriatrics, Center for Alzheimer Research, Department of Neurobiology, Care Sciences and Society, Karolinska Institutet, Neo 7th floor, 141 83 Stockholm, Sweden; 2 Department of Neurology, Karolinska University Hospital, Stockholm, Sweden; 3 Center for Neurocognitive Rehabilitation (CeRiN), CIMeC, University of Trento, Trento, Italy; 4 NIMTlab-Neuroimaging and Innovative Molecular Tracers Laboratory, University of Geneva, Geneva, Switzerland; 5 German Center for Neurodegenerative Diseases (DZNE), Rostock, Germany; 6 LANE—Laboratory of Alzheimer’s Neuroimaging and Epidemiology, IRCCS Istituto Centro San Giovanni di Dio Fatebenefratelli, Brescia, Italy; 7 Faculty of Medicine, University of Cologne, Cologne, Germany; 8 German Center for Neurodegenerative Diseases (DZNE), Bonn/Cologne, Germany; 9 Institute of Neuroscience and Medicine (INM-2), Molecular Organization of the Brain, Research Center Jülich, Jülich, Germany; 10 Clinical Memory Research Unit, Department of Clinical Sciences Malmö, Lund University, Lund, Sweden; 11 Memory Clinic, Skåne University Hospital, Malmö, Sweden; 12 Alzheimer Center Amsterdam, Department of Neurology, Amsterdam Neuroscience, Vrije Universiteit Amsterdam, Amsterdam UMC, Amsterdam, Netherlands; 13 Department of Clinical Memory Research, Lund University, Lund, Sweden; 14 Memory Clinic, University Hospital, Geneva, Switzerland; 15 Nuclear Medicine and Molecular Division, Geneva Medical Hospital, Geneva, Switzerland; 16 Theme Inflammation and Aging, Karolinska University Hospital, Stockholm, Sweden

**Keywords:** Alzheimer’s disease, Strategic roadmap, Biomarker-based diagnosis, Tau PET, THK, PBB3

## Abstract

**Purpose:**

The research community has focused on defining reliable biomarkers for the early detection of the pathological hallmarks of Alzheimer’s disease (AD). In 2017, the Geneva AD Biomarker Roadmap initiative adapted the framework for the systematic validation of oncological biomarkers to AD, with the aim to accelerate their development and implementation in clinical practice. The aim of this work was to assess the validation status of tau PET ligands of the THK family and PBB3 as imaging biomarkers for AD, based on the Biomarker Roadmap methodology.

**Methods:**

A panel of experts in AD biomarkers convened in November 2019 at a 2-day workshop in Geneva. The level of clinical validity of tau PET ligands of the THK family and PBB3 was assessed based on the 5-phase development framework before the meeting and discussed during the workshop.

**Results:**

PET radioligands of the THK family discriminate well between healthy controls and patients with AD dementia (phase 2; partly achieved) and recent evidence suggests an accurate diagnostic accuracy at the mild cognitive impairment (MCI) stage of the disease (phase 3; partly achieved). The phases 2 and 3 were considered not achieved for PBB3 since no evidence exists about the ligand’s diagnostic accuracy. Preliminary evidence exists about the secondary aims of each phase for all ligands.

**Conclusion:**

Much work remains for completing the aims of phases 2 and 3 and replicating the available evidence. However, it is unlikely that the validation process for these tracers will be completed, given the presence of off-target binding and the development of second-generation tracers with improved binding and pharmacokinetic properties.

## Introduction

In the past few years, there has been a boost in the development of imaging and fluid biomarkers for tau pathology, one of the neupathological hallmarks of Alzheimer’s disease (AD). Regarding imaging, a wide variety of PET ligands for tau was developed and an extensive amount of data from clinical studies has been published internationally from different research groups in a relatively short period of time (for a detailed review see [1]). However, whether these results support the validity of those tau biomarkers for use in a clinical setting remains to be assessed systematically. Such an assessment is of particular interest at the moment, given the recent US Food and Drug Administration (FDA) approval for clinical use of one of the developed ligands targeting tau pathology (i.e., AV-1451, aka Flortaucipir or Tauvid).

In 2017, a methodological 5-phase framework for the systematic assessment of biomarker validation has been imported from oncology [2] and adapted to AD [3]. Within this Biomarker Roadmap initiative, we had assessed the clinical validation status of all well-consolidated biomarkers at the time for a specific context of use, namely improved clinical diagnosis in patients presenting to memory clinics with mild cognitive impairment (MCI) [4]. These biomarkers included the episodic memory assessment [5], cerebrospinal fluid (CSF) measures [6], medial temporal atrophy [7], ^18^F-fluorodeoxyglucose (FDG) PET [8], amyloid-β ligands on PET [9], and ^123^I-ioflupane brain single-photon emission tomography and ^123^I-metaiodobenzylguanidine (MIBG) cardiac scintigraphy [10].

The present systematic review aims to apply the developed methodological 5-phase framework of the Biomarker Roadmap initiative—in its 2020 update that aimed to align it with the current research criteria [11] and accommodate tau biomarkers (Boccardi et al., in this Issue doi: 10.1007/s00259-020-05120-2)—for assessing the validation status of the tau PET ligands of the THK family (i.e., THK5117, THK5317, THK5351) and PBB3 for clinical use. Separate reviews assess the clinical validity of the other available tau PET ligands and measures of tau in CSF and plasma (Wolters et al.; Leuzy et al.; Bischof et al.; Ashton et al., in this Issue, doi: 10.1007/s00259-020-05118-w; 10.1007/s00259-020-05156-4).

## Methods

### Target

This literature review investigates the clinical validation status of tau PET ligands as AD biomarkers, in accordance with the Biomarker Roadmap initiative [3, 4] 2020 update (Boccardi et al., in this Issue). The context of biomarker use entails the accurate diagnosis of patients with MCI referred to memory clinics for cognitive complaints, which are attributed to a possible sporadic, and not autosomal dominant, dementing neurodegenerative disorder. The studies of ligands of the THK family (i.e., THK5117, THK5317, THK5351) and PBB3 that were eligible for this review used as reference standard for the biomarker-based diagnosis either AD histopathological examination when available, amyloid-β biomarker positivity, or development of incidental AD dementia during a follow-up interval of at least 2 years. Thus, eligible studies for assessing the clinical validity of the biomarker were both prospective longitudinal and cross-sectional designs. For the aims of the review, only ligands of the THK family and PBB3 were taken into consideration. The evidence for other ligands and the available tau bio-fluid markers is discussed elsewhere (Wolters et al.; Leuzy et al.; Bischof et al.; Ashton et al., in this issue).

### Glossary

#### Neuropathological diagnosis of Alzheimer’s disease

The definite diagnosis of AD is based on the presence of extracellular amyloid-β plaques and aggregates of hyperphosphorylated tau in neurofibrillary tangles in the brain of the affected individuals, independently from the clinical expression of cognitive symptoms. The presence of aggregates of amyloid-β and tau is often associated to the AD-pattern of medial temporal and temporoparietal neurodegeneration.

#### Clinical diagnosis of Alzheimer’s disease dementia

According to the classical criteria, as defined by the National Institute of Neurological and Communicative Disorders and Stroke and the Alzheimer’s disease and Related Disorders Association (NINCDS-ADRDA), AD can be diagnosed in the clinic at the dementia stage of the disease with two levels of certainty, namely possible and probable AD [12]. Notably, because of the imperfect accuracy of purely clinical criteria, a percentage of cases with a clinical diagnosis of AD might suffer from non-AD pathology since the diagnostic gold standard remains the histopathological examination. The most recent research criteria support that the use of biomarkers, especially those targeting amyloid-β pathology, could increase the diagnostic certainty that the basis of the clinical dementia syndrome is the AD pathophysiological process (i.e., AD dementia diagnosis; [13, 14]).

#### Mild cognitive impairment

This diagnosis refers to individuals with an acquired objective cognitive impairment but without, or with subtle functional disability. Representing a clinical syndrome, it encompasses cases progressing to AD (about 50%) or non-AD dementia (about 10–15%; [15–17]), and cases that are not deteriorating further cognitively over time (about 35–40%). MCI cases positive to AD biomarkers can be defined as prodromal AD or MCI due to AD based on research diagnostic criteria [14, 18]. The diagnosis of AD at the MCI stage represents the focus of the present review.

#### Non-AD neurodegenerative disease

This term refers to the large spectrum of neurodegenerative disorders considered for the differential diagnosis (e.g., the variants of frontotemporal lobar degeneration, dementia with Lewy bodies, hippocampal sclerosis, limbic-predominant age-related transactive response DNA-binding protein of 43 kDa (aka TDP-43) encephalopathy, primary age-related tauopathy (aka PART), argyrophilic grain disease).

### Conceptual framework

Details on the conceptual framework have been extensively discussed [3]. For each phase/aim, different strings were used to detect relevant studies, which were selected following PRISMA guidelines (see Online Resource for strings and PRISMA results). For all included studies, relevant information about study design, methods, and results was recorded.

#### Phase 1

This phase includes preclinical exploratory studies on the rational for using tau PET ligands of the THK family and PBB3 for detecting tau pathology in AD. The gold-standard for phase 1 studies is histopathological examination.

#### Phase 2

Phase 2 studies investigate the diagnostic accuracy of tau PET ligands of the THK family and PBB3 to distinguish patients with clinical diagnosis of AD from controls. Phase 2 studies are meant to define the clinical assay, to allow reliable assessment, and to identify the effect of confounders affecting the threshold for positivity in both patients and controls (e.g., age, gender, apolipoprotein ε4 status, education or comorbidities).

#### Phase 3

Phase 3 studies assess the biomarker ability to detect the disease at its earliest possible stage, namely MCI for this specific effort, in well-controlled experimental samples. Phase 3 studies aim to define criteria for positivity, to compare the diagnostic performance with other biomarkers, and to assess the diagnostic value of combinations of biomarkers, in view of defining a biomarker-based algorithm. These are normally prospective, longitudinal studies that distinguish between MCI individuals that deteriorate cognitively over time in the AD continuum (i.e., AD dementia) from those remaining cognitively stable or being diagnosed with other non-AD neurodegenerative diseases at follow-up. However, given the relative absence of such studies in the field of tau PET imaging to date, we even assessed at this phase separately studies with MCI patients, where the gold standard was not the follow-up assessment but the cross-sectional amyloid-β biomarker status of the patients, in accordance with the existing research criteria. Nonetheless, the cross-sectional amyloid-β biomarker status represents only a construct validity for this phase and the level of evidence that it provides is lower than the follow-up studies, given the inherent limitations of validating a new biomarker to a non-perfect existing clinical marker [19].

#### Phase 4

Phase 4 studies assess the performance of tau PET ligands of the THK family and PBB3 in representative patient cohorts from memory clinics. The biomarker itself is used to support a clinical diagnosis to patients with MCI who are subsequently treated based on this tau PET-supported diagnosis. They are meant to quantify the benefit of tau PET-based early detection, and of practical feasibility and protocol compliance. Preliminary evidence about costs is an additional aim, in view of dedicated studies in phase 5.

#### Phase 5

Phase 5 studies evaluate the impact of the tau PET-based diagnosis on society (e.g., cost-effectiveness relative to clinically meaningful outcomes).

### Evidence assessment

The fulfillment of each validation step from phase 1 to phase 5 has been assessed consistently with the 2017 Biomarker Roadmap initiative [3], update 2020 (Boccardi et al., in this issue). As such, primary and secondary aims for each phase were rated as follows: fully achieved, partly achieved, preliminary evidence, not achieved or unsuccessful, as defined below. To facilitate the assessment and make it transparent to the readers, the data used to define the degree of fulfillment for each aim are reported and summarized in tables accessible online (see Online Resource).

Fully achieved: available scientific evidence, successfully replicated in properly powered and well-designed studies.

Partly achieved: the available evidence is not sufficiently replicated, or samples are not adequately powered, or studies are faulted with major methodological limitations.

Preliminary evidence: only preliminary evidence is available.

Not achieved: studies are not yet performed at the time of the review.

Unsuccessful: available scientific evidence shows a failure for the biomarker in achieving the aim. Findings in the subsequent Biomarker Roadmap phases should be interpreted with caution.

### Papers search and selection

For each phase, we performed systematic literature searches for studies investigating the previously mentioned aims. We searched PubMed and Embase databases for relevant studies. The search was conducted on 16.05.2020. General search strings (“THK-5117” OR “THK-5317” OR “THK-5351” OR “THK5117” OR “THK5317” OR “THK5351” OR “PBB3”) were used to identify articles about the relevant tau PET ligands, which were combined with specific search strings for every phase, as detailed in the Online Resource. Only articles written in English were included. These searches were supplemented by the admission of relevant evidence based on personal knowledge or from the reference lists of pertinent articles. After excluding the duplicates, all titles identified by search strategies were assessed for relevance based on their abstract independently by two reviewers, the authors KC and CF. All resulting records were assessed for eligibility in their full text by the same authors (KC, CF). The reasons for exclusion and the number of finally retained papers for each phase/aim are reported according to the PRISMA guidance [20] on the Online Resource (https://drive.switch.ch/index.php/s/4reUTSuqNZHyIC8).

## Current clinical validity of tau PET THK and PBB3 imaging

- Systematic review searches

Studies identified for each phase/aim are reported in the respective PRISMA flow charts (Online Resource).

b) Clinical validity of ligands of the THK family and PBB3

**Fig. 1:**
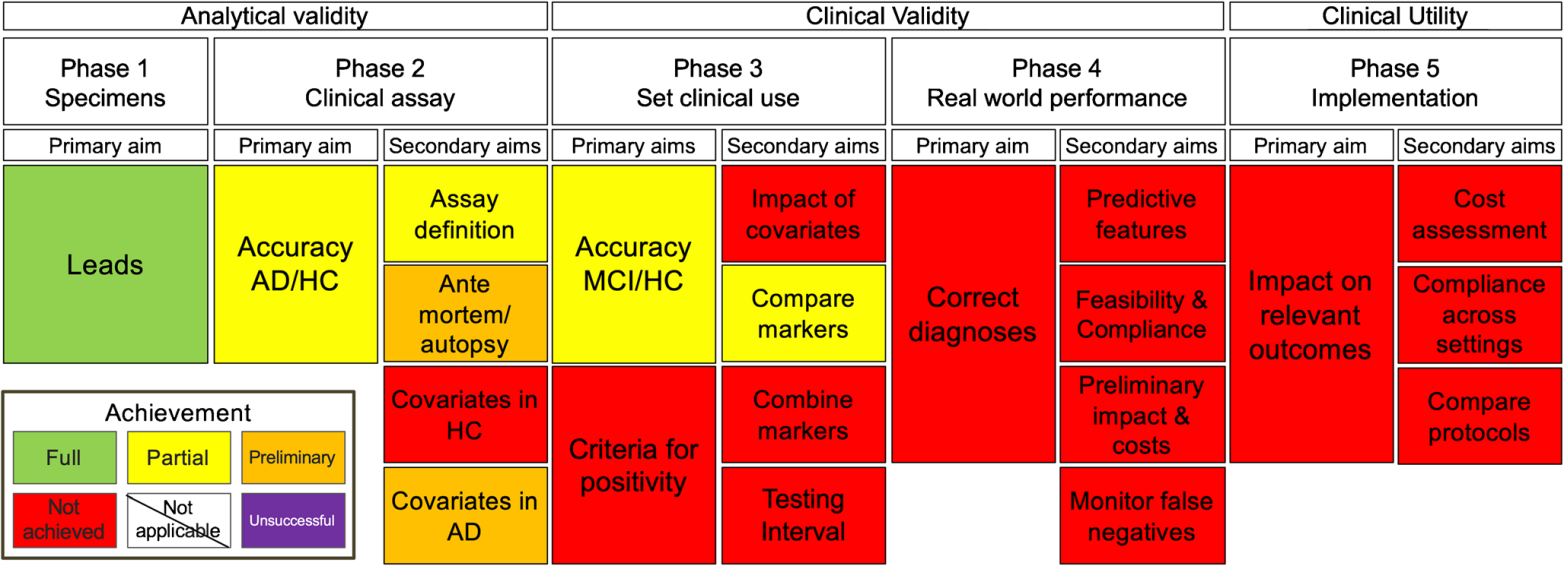
Synopsis of the clinical validity of tau PET ligands of the THK family as adapted from an oncology framework [2, 4]

**Fig. 2:**
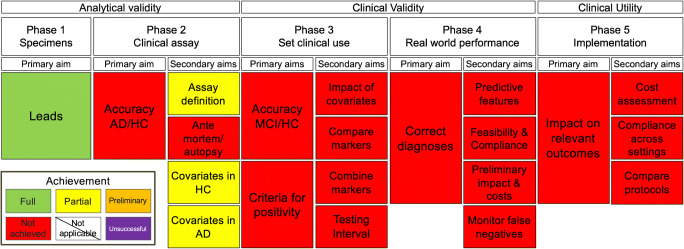
Synopsis of the clinical validity of PBB3 PET as adapted from an oncology framework [2, 4]

Figures 1 and 2 summarize the current state of tau PET ligands of the THK family and PBB3, respectively, as per our methodological framework.

### Phase 1: preclinical exploratory studies

#### Primary aim: to identify leads for potentially useful biomarkers

PET ligands of the THK family (i.e., THK5117, THK5317, THK5351) and PBB3 have shown in vitro high affinity in the nanomolar range for a wide span of different conformations of tau aggregates, and selectivity for tau over amyloid-β aggregates [21–27]. When autoradiography in brain tissue was performed, the binding pattern of those ligands resembled closely the binding pattern of standard tau-specific antibodies. All ligands showed pre-clinically favorable pharmacokinetics, with the enantiomerically pure most recent ligands of the THK family (i.e., THK5317, THK5351) showing improved properties compared to their racemic forms. Both the ligands of the THK family and PBB3 have shown evidence of binding to non-tau targets (i.e., off-target binding) predominantly in the basal ganglia and thalamus for the THK ligands, and the basal ganglia and vascular structures (e.g., choroid plexus, dural venous sinuses) for PBB3 (for a detailed review see [1, 28]). This aim was considered fully achieved for the ligands of the THK family and PBB3.

### Phase 2: clinical assay development for Alzheimer’s disease pathology

#### Phase 2: primary aim: to estimate true positive and false positive rates or receiving operating characteristics curves (ROC) for the assay and to identify the discrimination accuracy between subjects with and without the disease

Three small-scale studies (*n* = 10–18) have assessed the accuracy of the ligands of the THK family in discriminating between clinically diagnosed AD patients and healthy controls (HCs). For THK5117, an effect size of Cohen’s *d* = 3.05 was reported [22]. In agreement to the latter study, for THK5317 and THK5351, accuracies as high as 99% and 96% were reported, respectively [29, 30]. The reported accuracies were dependent of the region of interest selected for each study, with areas of the temporal cortex being consistently reported as having the highest accuracy in both studies. No studies report formally the accuracy of ligands of the THK family to discriminate between clinically diagnosed AD patients and non-AD neurodegenerative disease although the evidence so far points towards a different regional pattern of ligand for both THK5317 and THK5351 binding in AD compared to other neurodegenerative diseases [29, 31–46].

To the best of our knowledge, no study has yet attempted to assess the accuracy of PBB3, although the evidence so far imply a good accuracy for discrimination of HCs and clinically diagnosed AD patients and a different regional pattern of binding for non-AD neurodegenerative diseases [27, 47–52].

Given the consistent findings of the small-scale studies, this aim was considered partly achieved for ligands of the THK family and not achieved for PBB3. We underline however that phase 2 is aimed to demonstrate that the assay does detect the anomaly of interest, thus performing studies using pathology as gold standard is of paramount importance and should be considered a short-term priority.

#### Phase 2: secondary aim 1: to optimize procedures for performing the assay and to assess the reproducibility of the assay within and between laboratories

The binding of THK5317 and THK5351 has shown strong correlation when the ligands were injected in the same individuals [53]. Low test-retest variability for THK5317, absence of known brain-penetrating metabolites for THK5351, and robust quantification of the binding of both ligands were reported even with simplified reference region-based approaches with or without the use of structural imaging for region of interest identification [24, 29, 30, 53–59]. The effect of partial volume effect correction methods in the binding quantification remains unclear for THK5351, while for THK5317 partial volume effect correction offers better discrimination between diagnostic groups in small regions of interest (e.g., hippocampus, anterior cingulate gyrus) [29, 60].

For PBB3 a radiolabeled metabolite, which crosses the blood-brain barrier, has been identified [61, 62], although a robust quantification of the ligand binding could be achieved with various reference region-based approaches, despite the metabolite signal [63, 64].

Overall, this aim was considered as partly achieved for ligands of the THK family and PBB3.

#### Phase 2: secondary aim 2: to assess the consistency of the ante-mortem binding of the ligand and the histopathological measurements of tau pathology

For ligands of the THK family, two independent studies have assessed the consistency of the ligand binding in vivo with histopathological evidence of tau. Harada et al. reported in a single AD case, which was assessed in vivo with THK5351 PET imaging and post-mortem with histopathology, that the THK5351 binding was associated with both the loads of tau pathology and monoamine oxidase B (MAO-B) enzyme; off-target binding to MAO-B has been identified by translational studies for several of the developed tau PET ligands [65]. On the contrary, Leinonen et al. used a different design and assessed the agreement of in vivo THK5317 binding and histopathological evidence of tau in biopsy material of patients with normal pressure hydrocephalus, without a clinical diagnosis of AD [66]. The authors reported no agreement between the two, although they acknowledged several limitations that this design raises. Given the major limitations of both studies, only preliminary evidence exists for ligands of the THK family, while no studies exist for PBB3 and therefore the aim was considered as not achieved for this ligand.

#### Phase 2: secondary aim 3: to assess covariates (such as gender, age) associated with biomarker status or level in control subjects

To the best of our knowledge, no study has yet assessed the impact of covariates in the binding levels of control subjects for ligands of the THK family. Regarding PBB3, evidence suggests a higher binding of the ligand with higher age and lower educational attainment [47]. This aim was considered partly achieved for PBB3, based on evidence of one study addressing the research question, and not achieved for ligands of the THK family.

#### Phase 2: secondary aim 4: to assess covariates (such as gender and age) associated with biomarker status or level in diseased subjects

The impact of covariates such as gender and age has not yet been assessed for ligands of the THK family in AD patients. However, different AD clinical syndromes were associated with a different regional pattern of binding for the ligand THK5351 [67, 68], which might bias the individual biomarker status if the same regions of interest are used for all patients irrespective of the clinical symptomatology.

For PBB3, evidence suggests a lower binding of the ligand with higher age and higher educational attainment [47, 56]. The impact of different AD clinical syndromes has not been assessed for PBB3.

In summary, preliminary evidence exists for ligands of the THK family, as can be derived from studies on AD clinical syndromes, while the aim was considered partly achieved for PBB3, based on results of one study investigating this research question.

### Phase 3: prospective longitudinal repository studies

#### Phase 3: primary aim 1: to evaluate the biomarker ability in the detection of the disease at the earliest clinical stage (MCI due to AD) using conversion to AD dementia as the reference standard

A single small-scale monocentric study has, so far, investigated the accuracy of THK5317 in discriminating between amyloid-β positive cognitively impaired patients that will remain cognitively stable (considered being non-AD-related cognitive impairment) from those who deteriorated further cognitively to clinical AD dementia, with an average follow-up interval of 4 years [69]. According to the latter study, the accuracy of THK5317 in temporal regions of interest was excellent, up to 100%. To the best of our knowledge, no study has yet attempted to assess the accuracy of the other ligands of the THK family or PBB3. In summary, this aim was considered partly achieved for ligands of the THK family, based on evidence of one study, while not achieved for PBB3.

#### Phase 3: primary aim 2: to define criteria for a positive diagnostic test for MCI due to AD, in preparation of phase 4

This aim had not been achieved at the time of writing this review.

#### Phase 3: secondary aim 1: to explore the impact of relevant covariates on the biomarker discrimination abilities at the MCI stage

This aim had not been achieved at the time of writing this review.

#### Phase 3: secondary aim 2: to compare the different biomarkers available to select the most promising ones

The only available study comparing the prognostic accuracy of THK5317 with that of FDG PET, tau assessment in the CSF, clinical atrophy rating on MRI, and neuropsychological measures reported that the THK5317 showed far greater accuracy than all other markers [69]. To the best of our knowledge, no study has yet attempted to assess the accuracy of the other ligands of the THK family or PBB3. This aim was considered partly achieved for ligands of the THK family, based on evidence of one study, while not achieved for PBB3.

#### Phase 3: secondary aim 3: to develop and validate diagnostic algorithms combining biomarkers for an optimal performance

This aim had not been achieved at the time of writing this review.

#### Phase 3: secondary aim 4: if repeated testing is needed, to determine a biomarker-testing interval in prevision for phase 4 studies

This aim had not been achieved at the time of writing this review.

### Phase 4: prospective diagnostic studies

The aims of this phase had not been achieved at the time of writing this review.

### Phase 5: disease-control studies

The aims of this phase had not been achieved at the time of writing this review.

## Discussion

With this work, we assessed the clinical validity of the PET ligands of the THK family and PBB3 as biomarkers of brain tauopathy according to the 5-phase framework proposed by the Biomarker Roadmap initiative [4] and its 2020 update (Boccardi et al., in this issue). Ligands of the THK family discriminate well between HCs and patients with clinically diagnosed AD (Phase 2) and recent evidence suggest an accurate diagnostic performance for the THK5317 at the early MCI stage of the disease (phase 3). No formal evidence exists about neither phase 2 nor phase 3 primary aims for PBB3. Little evidence exists, so far, about most secondary aims of phases 2 and 3 for ligands of the THK family and PBB3. No evidence exists for phases 4 and 5.

Although several reviews about tau PET imaging have been published, no systematic reviews have been performed which would assess the validity of the clinical use of this biomarker in AD. The Biomarker Roadmap initiative assessed originally other biomarkers, but this effort is fully consistent with that validation methodology. This kind of work is necessary to coordinate efforts across independent research groups. Greater awareness of completed steps, research gaps, and priorities based on a sound consensual methodological framework might improve the cost-effectiveness of subsequent validation studies. This work should not be interpreted as an effort to promote the clinical use of the reviewed ligands but rather present in an objective, structured, and validated framework the available evidence for the existing tau biomarkers (tau PET ligands for imaging and tau measures in blood and CSF) in parallel reviews.

The ligands of the THK family have been the only ones from all developed tau PET ligands that were tested longitudinally in a phase 3 study at the time of writing this review [69]. The generalizability of these promising results is, however, subject to several gaps that remain to be filled as research priorities for assessing the clinical validity of the THK ligands: namely, the assessment of (1) the discriminative ability of the assay in pathology confirmed AD and HC samples for phase 2; (2) the accuracy of the biomarker to discriminate between AD and non-AD neurodegenerative disease in adequately powered studies; (3) the consistency of the ante-mortem and the histopathological measurements of tau pathology, which remains, so far, inconclusive; (4) covariates that are associated with the biomarker status in patients with AD and HCs; (5) the replicability of the available evidence about the biomarker’s diagnostic performance at the MCI stage; (6) criteria of biomarker positivity.

For the purposes of this review, we assessed TH5317 and THK5351 as a whole (THK family) summing up the results obtained from studies using either THK5317 or THK5351 (Fig. 1). This approach was chosen given the high structural similarity of the two ligands and the strong association of the binding of the two when injected in the same individual [53]. However, the main weakness of this generalizability of evidence is the reported differences between ligands in terms of pharmacokinetics and dynamic range (more favorable pharmacokinetics and wider range for THK5351 relatively to THK5317 [53]), and degree of off-target binding (higher for THK5351 relatively to THK5317 [70]) All these factors could affect the comparability of the validity of the two ligands in several aspects of the current framework, although no head-to-head comparisons of diagnostic accuracy have been performed. To address this issue, we reported separately in the text for each aim and subaim which of the two tracers was used to obtain the specific results, in order to allow the reader to critically appraise the available evidence for each ligand.

For PBB3, phase 2 studies assessing the accuracy of the biomarker to discriminate between clinically diagnosed AD patients and HCs or non-AD neurodegenerative diseases are still missing. Those studies, possibly validated by neuropathological assessment, remain the first research priority before one could continue with assessing other research priorities. PBB3 however has a number of limitations, namely (1) the short half-life with need for on-site production of the ligand (^11^C-labeled), (2) off-target binding signal, (3) a relatively narrow dynamic range of binding values [56], (4) the existence of brain penetrating metabolites that challenge the quantification of the binding, and (5) the sensitivity of the ligand to photoisomerization that limits the transportation and injection of the ligand in dark conditions.

A fluorinated derivative of PBB3 (^11^C-labeled) with somewhat different pharmacological properties has now been developed (i.e., ^18^F-APN-1607) and is intended to substitute the original ligand [71]. Hopefully, the longer half-life of ^18^F would allow the thorough investigation of the ligand and the emergence of replication studies, something that was limited with PBB3 since most of the evidence derives from the original developers of the ligand.

The interaction of tau PET ligands with non-intended targets, the so-called off-target binding, is so far challenging their validity, since several targets have been identified in in vitro, in vivo, and even in silico studies [1, 28]. With regard to ligands of the THK family, off-target binding signal has been attributed to binding to MAO-B. MAO-B is an enzyme that catalyzes the de-amination of neurotransmitters, mainly present in subcortical nuclei and it is implicated in a variety of normal and abnormal brain functions. Binding to MAO-B has been the main factor that halted the clinical interest on THK ligands, especially since second generation tau ligands were developed with improved binding and pharmacokinetic properties. The presence of off-target binding (to MAO-B and other non-tau targets) applies not only to ligands of the THK family, but even PBB3 and to the FDA approved ligand AV-1451 (aka, Flortaucipir or Tauvid). However, it is still unclear to what extent this off-target binding contributes to the in vivo signal of the different ligands. Thorough studies assessing the relationship between ante-mortem PET signal and histopathological measurements of those targets should be prioritized for shedding new light on this point.

Although our approach adhered to a sound methodology, rating degree of achievement for each aim should be based on a more thorough assessment of evidence, including examining various possible sources of bias (e.g., GRADE guidelines [72]). Our Online Resources, reporting data extraction for study features including possible risks of bias, are meant to help this development as a next step forward in a systematic assessment of the validation of AD biomarkers. Relative to the review results, we admitted studies with clinical reference standard or other evidence of construct validity; however, studies including pathology are still scarce and should be considered a priority for the proper completion of phase 2. Furthermore, another source of bias derives from the admission of evidence solely from peer-reviewed published studies. While the latter was done for ensuring the quality of reported evidence, one should acknowledge that much of the data of proprietary drugs are either published with delay or not published at all.
